# A Quarter-Century of Online Informatics Education: Learners Served and Lessons Learned

**DOI:** 10.2196/59066

**Published:** 2024-08-06

**Authors:** William Hersh

**Affiliations:** 1 Department of Medical Informatics & Clinical Epidemiology School of Medicine Oregon Health & Science University Portland, OR United States

**Keywords:** distance education, online learning, asynchronous education, biomedical and health informatics, learning, biomedical, health informatics, education, educational, educational technology, online program, online course, teaching, students

## Abstract

The value and methods of online learning have changed tremendously over the last 25 years. The goal of this paper is to review a quarter-century of experience with online learning by the author in the field of biomedical and health informatics, describing the learners served and the lessons learned. The author details the history of the decision to pursue online education in informatics, describing the approaches taken as educational technology evolved over time. A large number of learners have been served, and the online learning approach has been well-received, with many lessons learned to optimize the educational experience. Online education in biomedical and health informatics has provided a scalable and exemplary approach to learning in this field.

## Introduction

Twenty-five years ago, in early 1999, I made a decision that seemed like just another project at the time but turned out to profoundly affect the work of my career. This was a decision to investigate the interest and subsequently move my introductory biomedical and health informatics course in our graduate program at Oregon Health & Science University (OHSU) to an online format. It was just a single course, but would lead to profound new directions not only for my career but also for the graduate program I led.

In 1999, the internet and online educational environment were much different from that in the present. Although many people had wired internet connections of decent bandwidth at their work, broadband internet to the home was relatively new. There was no wireless connectivity to speak of. The online educational environment was similarly nascent at the time, and distance learning was viewed as a second-class method of education. A quarter-century later, we can look back at the learners who were served and the lessons learned.

## Prelude

The decision to move my introductory course online was driven by potential students expressing interest in the course being offered online. In early 1999, we gauged interest by conducting a mail survey of 500 random people each on the American Medical Informatics Association (AMIA) and Healthcare Information and Management Systems Society (HIMSS) listservs [1]. From the 288 surveys returned, there was definite interest in the course, its delivery on the World Wide Web, the need to have access by dial-up modem (not everyone had broadband internet in their homes), and content covering a range of topics similar to what was in the existing on-campus course I was already teaching. Although the OHSU School of Nursing and the Educational Communications Department had started pursuing distance learning, there was no institutional infrastructure to support our effort.

I had already been teaching my introductory course for over half a decade. In the early 1990s, I was relatively a new Assistant Professor at OHSU, where I had come after completing medical training at the University of Illinois Chicago and a National Library of Medicine (NLM)–funded postdoctoral fellowship at Harvard University. I was recruited to OHSU as part of a new academic informatics program funded by the Integrated Advanced Information Management Systems initiative of NLM [2]. My early focus, like most in informatics, was developing a research program.

However, I always had an interest in teaching and had the opportunity to develop an introductory informatics course in 1993 that was an elective in OHSU’s relatively new Master of Public Health program. The course initially consisted of lectures by half a dozen informatics faculty who were part of the program. Although others enjoyed lecturing on their areas of expertise, I had larger ambitions, and under the mentorship of the OHSU Provost at the time, Lesley Hallick, PhD, set out to launch a Master of Science (MS) in Medical Informatics program. OHSU had already been funded under the NLM T15 Training Grant program since 1992.

In 1996, OHSU received approval from the state of Oregon to launch our MS program. We welcomed 7 entering students, all of whom graduated in 1998 or 1999. The introductory course that was previously identified as Public Health 549 now became Medical Informatics (MINF) 510. The course was taught with 3 hours of lecture and other classroom activities once a week. Other courses were developed for the program, including clinical decision support, computer science, and organizational behavior and management. One interesting finding for me was that many of our students aspired to assume informatics careers outside of the main path for the field in the 20th century, which was as an academic researcher that was typically grant-funded. A number of them, however, saw health care institutions, industry, and other sites beginning to hire operational informatics professionals. Many were adult learners who wanted to learn more but could not quit their day jobs nor uproot their families to places like Oregon to pursue in-person education.

## Initial Course

Before 1999, people were inquiring about the availability of my course and our program online. The prospect of adopting this technology was not daunting, but having very little experience and understanding of online education was. I had no formal training in education. I taught as many faculty did in the 1990s—lecturing with overhead transparencies that gave way to PowerPoint slides along with assigned readings, homework exercises, and a course project that was typically a term paper on a topic of the student’s interest.

Nonetheless, after the survey of interest conducted in 1999, I decided to offer a version of my introductory course in an online format. I did not abandon the in-person classroom, but rather offered a second section of the course, with my standing up in front of class lecturing replaced with recorded lectures that were made available online. I had started to learn some about what were considered state-of-the-art approaches to online lectures for the late 20th century. One key point was to be cognizant of bandwidth, as many people still connected to the internet via a telephone modem, especially from home. As such, we needed to be careful that all materials, including the recorded lectures, could be delivered over telephone modem speeds, that is, 28-56 Kb/s modems. This precluded the use of video, high-resolution images, or other bandwidth-intensive media.

**Figure 1:**
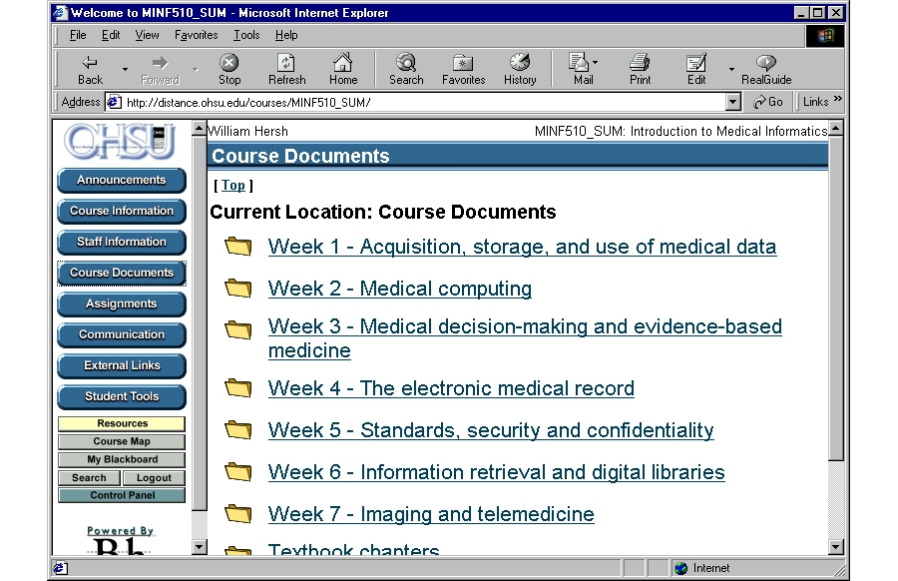
Course outline in the Blackboard learning management system. MINF: Medical Informatics; OHSU: Oregon Health & Science University.

**Figure 2:**
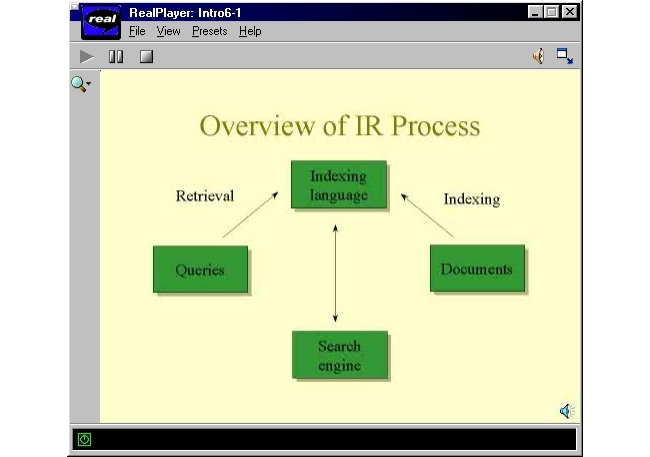
Lecture in RealMedia format displaying the elements of information retrieval systems. IR: information retrieval.

Textbox 1 shows the weekly course outline for the course at the time, which was later moved to an online format. The topics covered then are still pertinent in the present, but many new ones have come to the fore. Figures 1 and 2 show the learning management system (LMS) and lecture screenshots from the first course offering.

**Course outline circa 1999.**

- Acquisition, storage, and use of medical data
- Medical computing
- Medical decision-making and evidence-based medicine
- The electronic medical record
- Standards, security, and confidentiality
- Information retrieval and digital libraries
- Imaging and telemedicine
- Artificial intelligence and decision support
- Computer networks and the internet
- Nursing and consumer health informatics
- Evaluation of medical informatics applications

Fortunately, I already had the lecture slides for the course; therefore, I was able to add narration to them, that is, voice-over PowerPoint. Another adage at the time was that online lectures be parsed into segments typically of about 15-20 minutes each. As such, I broke my PowerPoint lecture files into segments and I soon came to learn how much narration I typically used per slide, which to this day is an average of about 90 seconds.

The most common format for the delivery of media in the early days of the World Wide Web was RealMedia (RealNetworks). Producing RealMedia files required recording the entire lecture and compiling the output using a tool called Real Presenter. Our department had no LMS before we started this journey, and the leading system at the time was Blackboard (Anthology), which we installed on a Unix server in our department. Fortunately, the hardware and software costs for this were modest at the time.

The initial course was successful by metrics of student performance on homework assignments and examinations being comparable to those of on-campus students, although they were admittedly different audiences [1]. The 15 students completing the course expressed great satisfaction and were interested in taking additional courses. Another interesting finding was that the volume of the interactive messages in the discussion forum vastly exceeded the amount of discussion that took place in the on-campus course, showing that online courses could be at least as interactive as those taught in-person.

## Additional Courses and Development of Educational Programs

There was enough demand for the online course that I started offering it every academic quarter, which I have done continuously since 1999. The success of the first course led us to develop our first credential, a Graduate Certificate, which was launched in 2000 [3]. This allowed other faculty to move their courses to an online format. We initially did not think that there would be interest in or a market for a full MS program online. However, many students in the Graduate Certificate Program did express an interest in obtaining an online MS degree, thus leading to the launch of that program in 2002.

When our online MS program was launched, we believed that students should spend some time on our campus. We did this by teaching some courses in a short-course format, where the course was taught intensively for 3-5 days, often with additional activity before or after the on-campus intensive. These courses were typically offered during the summer when students had more flexible schedules and the weather was nicer in the Pacific Northwest.

I also moved other courses that I taught to an online format. I taught a course on information retrieval, the area of my research, and moved that online in 2002. I also started teaching a new course on evidence-based medicine based on the McMaster University model [4], exclusively online. Unlike the other courses, the latter only had modest amounts of online lecture material and mainly consisted of students critically appraising journal papers and presenting them to their classmates.

All through this time, we maintained our on-campus graduate program. We launched our PhD program in 2003, which funded students mainly from our NLM T15 grant. Our first PhD graduate was Adam Wright, who is now a professor in the Department of Biomedical Informatics (DBMI) at Vanderbilt University. Another graduate from the program at the time was Peter Embi, MD, MS, who is now Chair of the Vanderbilt DBMI.

Around this time, we made some other changes in our graduate program. One was to bifurcate the program into 2 tracks, which are now called majors. The original track/major is now called Health & Clinical Informatics (HCIN), while the newer track/major is now called Bioinformatics/Computational Biomedicine (BCB). At this time, we also changed the prefix name of our courses from MINF to Biomedical Informatics (BMI), that is, my introductory course became BMI 510.

## Evolution of Tools and Platforms

As the internet evolved, so did the availability of new and improved tools for developing and disseminating content. In 2004, we began producing lectures in the Adobe Flash format, initially via the Adobe Presenter tool. In 2009, we moved to a different tool for producing Flash content, called Articulate Presenter. This tool had a new feature of allowing each slide to be recorded individually and then exported into a single Flash file. This turned out to be a major time-saver, as only new slides or those with updated content needed to recorded again. In addition, the output of Articulate Presenter added some additional navigational tools, such as a list of the slides and incorporation of notes, which were used for accessibility. In the mid-2010s, as the use of Flash was replaced by HTML5, Articulate Presenter allowed output in that format, allowing us to transition content from Flash to HTML5 without having to change any underlying source material.

I made a personal transition in 2012 when returning to using the Apple Macintosh after a hiatus using Microsoft Windows computers from 1995 to 2012. A big hesitancy for switching was my use of Articulate Presenter, which only ran under Windows. However, Apple had recently moved to Intel chips, with the ability to run Windows. Even though it was clunky, I was able to maintain the use of Articulate Presenter by using the virtual-machine software Parallels. Many users of Articulate Presenter, mostly academicians, requested for the software to have a native Mac version, but Articulate never developed one. Additional dissatisfaction with Articulate was caused by its movement to an expensive subscription model.

By 2021, I had another reason to move on from Articulate Presenter, which was when Apple moved from Intel to ARM (Advanced Reduced Instruction Set Computing Machines) processors. Fortunately, the slide narration and export options of PowerPoint itself had improved markedly, and I was able to produce narrated lectures exclusively with PowerPoint. I exported the slideshow as an MP4 video and uploaded the file to Echo360, which had been adopted by OHSU as a media server.

Another transition was OHSU adopting an institutional LMS in 2007. This enabled our department to not have to maintain our own LMS or server for it. The university adopted Sakai, an open-source LMS. OHSU chose the option of a vendor to run Sakai in their data center. Sakai provides most of the features seen in an LMS, including organization of courses, provision of content or links to it, multiple choice questions, discussion forums, gradebooks, and linkage to student management systems.

## AMIA 10x10 Program

Another major event in my online teaching journey was the AMIA 10x10 (ten by ten) program. In 2005, when Charles Safran, MD, was President of AMIA, he was convinced that the United States needed more people, especially physicians and nurses, trained in informatics [5]. Dr Safran advocated that the United States needed at least 1 physician and 1 nurse trained in informatics in the nearly 6000 hospitals in the United States. This led him to ask a number of educational program directors, including myself, how much capacity they had to increase the size of their programs. Although most of them said perhaps they could increase 2- or 3-fold, my reply to him was “all of them.” This was because I knew that distance learning was very scalable, and with enough lead time could be scaled up with faculty to support much larger numbers of learners.

At that time, AMIA was looking to develop some sort of introductory course in BMI. However, the prices quoted to them by vendors were beyond AMIA’s means. As I already had my online introductory course from our graduate program, I proposed to AMIA and Don Detmer, MD, MS, its President and Chief Executive Officer, a repackaging of my online course. I proposed the name 10x10 based on Dr Safran’s stated need of 1 physician and nurse trained in informatics in 5000+ US hospitals and set a goal for doing so by 2010. Because the course already existed, we were able to quickly put in place a memorandum of understanding between OHSU and AMIA, which was based on an agreement of mutual nonexclusivity: OHSU would maintain the ownership of the 10x10 course content (for use in other programs, including our graduate program) and AMIA could offer other 10x10 courses. Dr Detmer later called 10x10 one of the most successful AMIA initiatives ever undertaken.

In the summer of 2005, the first offering of 10x10 was launched, with 51 people enrolled [6]. Unlike our graduate course that was only online, an in-person session would bring course participants together at the AMIA Annual Symposium. A total of 44 people completed the first offering, and almost all of them showed up for the first face-to-face session in November.

One of the participants in the original 10x10 course was Paula Otero, MD, a pediatrician from Hospital Italiano de Buenos Aires in Argentina. After the course ended, she proposed an interesting idea: translating the course into Spanish [7]. This started a very productive collaboration that resulted in the later awarding of an informatics training grant from the National Institutes of Health Fogarty International Center. This allowed Dr Otero to further develop her online offering in Spanish, while also sending faculty for informatics fellowship training to OHSU.

The 10x10 course was successful by many metrics, attracting large numbers and achieving strong satisfaction from students completing it [8]. We structured the course such that those who received a grade of B or better on an optional final examination could obtain an academic credit in our program at OHSU. This would also then allow them to enroll in the OHSU Graduate Certificate or MS program (or obtain credit and transfer it to other graduate programs). About 10%-15% of those completing the 10x10 course have gone on to further study in the field.

## Health Information Technology for Economic and Clinical Health Era

As my own interest in informatics education and training grew, I began to develop a research interest in the informatics workforce [9]. Unable to find any definitive data about the workforce, I came across the HIMSS Analytics Database. Although not an optimal source of data to answer questions about the workforce, I was able to use the database to gain the first estimate of the size of the workforce and its potential growth, as hospitals moved to higher levels of electronic health record adoption [10]. In addition to presenting this work at the AMIA Annual Symposium in 2008, I also had the opportunity to present it in Capitol Hill earlier that year, which was fortuitous due to the imminent Great Recession coming, with the US government creating the American Recovery and Reinvestment Act that aimed to stimulate the economy and create jobs.

A major part of the American Recovery and Reinvestment Act was the Health Information Technology for Economic and Clinical Health (HITECH) Act, with investment over US $30 billion to facilitate the adoption and meaningful use of the electronic health records [11]. In part because of my workforce research, a US $118 million workforce development program was made part of the HITECH investment. OHSU played a large role in the grants that were competitively awarded in 2010 by the workforce development program, including serving as the National Coordination and Dissemination Center for the health information technology curriculum that was funded for development [12].

During and after HITECH, I continued to provide leadership around informatics education and how it advanced other careers in the field. I was also a leader in the new clinical informatics physician subspecialty [13], being appointed by AMIA to direct the Clinical Informatics Board Review Course (CIBRC), which was offered in time for the first board examination in 2013. I recruited an excellent team to help teach the course, including Thomas Payne, MD; Bimal Desai, MD; and Diane Montella, MD. As I was eligible for the board examination myself (even though I was no longer clinically active, I had completed my internal medicine residency in the era of lifetime certification), I took the examination and even passed! The next year, I laid the groundwork at OHSU to establish one of the first 4 Accreditation Council for Graduate Medical Education–accredited fellowships for the new subspecialty, which launched in 2015 under the leadership of Vishnu Mohan, MD [14].

## The Last Frontier: Medical Education

Although I had been interested in informatics education for others besides informaticians dating to the 1990s, I was never able to make headway in obtaining any significant content into the OHSU Doctor of Medicine (MD) educational program. Finally, in 2012, with the arrival of a new supportive Senior Associate Dean for Medical Education, George Mejicano, MD, MS, the door was opened for me and my colleagues in our department. We were also aided by being one of the 11 institutions awarded grants by the American Medical Association to accelerate change in medical education. Our work in clinical informatics education for medical students led to a 2014 paper that laid out the competencies for medical students, although as noted in the paper, could actually be applied to all health care students and professionals [15]. Along with colleagues at OHSU, we began to implement informatics education in the MD curriculum. We have started working with local colleagues in nursing, biomedical basic sciences, and public health to add informatics to their curricula.

Another medical education opportunity arose at the start of the COVID-19 pandemic. As the pandemic resulted in many medical students being displaced from ward and clinic rotations, there was a need at OHSU and other medical schools to find virtual education opportunities. It was easy to quickly adopt the introductory course curriculum to a medical school-style block course. Some colleagues from other medical schools learned of the availability of the online course and asked if it could be used at their institutions. In the first few months of the pandemic, the course was delivered to both OHSU students (3 offerings to a total of 44 students) and non-OHSU students (8 offerings to a total of 178 students). The excess workload required me to stop offering the course outside OHSU, but the course continues as a medical student elective at OHSU.

## Learners Served

I have been gratified that my online teaching has attracted so many learners over the years. Since the inception of the introductory course (MINF/BMI 510) in 1996, 1683 students have completed the course in its on-campus and online versions. Since the introduction of the AMIA 10x10 course in 2005, 3253 people have completed the OHSU offering of the course through early 2024. The medical student elective at OHSU continues to attract about 30 medical students per year. The OHSU BMI graduate program has 984 alumni, with 499 Graduate Certificates, 444 MS degrees, and 41 PhD degrees awarded.

There are now about 3000 physicians who are board-certified in the clinical informatics subspecialty. A large majority of those passing the exam have taken the CIBRC. I was gratified in 2016 when attending a reception for physicians at the Epic Advanced User’s Group meeting in Madison, WI, and finding that at least half of those I spoke with had taken one of the above educational experiences I had developed. It is not infrequent to find myself walking through an airport or another venue and have someone introduce themselves as having taken a course of mine, usually 10x10.

Although the main 10x10 course has been offered through AMIA, we have partnered with other organizations to offer versions tailored to individuals within them. The online curriculum content does not change, but rather, the student composition, discussion, related activities, and in-person sessions are modified to meet the needs of those organizations. Table 1 shows the partnerships with the number of course offerings and students completing them for both United States and international partners. The most prominent international collaboration has been with Gateway Consulting of Singapore, and the course continues to be cotaught with KC Lun, PhD.

**Table 1:** Number of 10x10 offerings and students completing the course with United States and international partners.

Organization			Offerings (n=114), n		Completed (N=3253), n
**United States**					
	American Medical Informatics Association (AMIA)	48		2159	
	American College of Emergency Physicians (ACEP)	16		237	
	American College of Physicians (ACP)	1		25	
	Association of Nutrition and Dietetics (AND)	7		126	
	Centers for Disease Control (CDC)	1		18	
	California Healthcare Foundation (CHCF)	1		16	
	Mayo Clinic	2		87	
	New York State Academy of Family Physicians	3		22	
	Scottsdale Institute (SI)	1		15	
	Society for Technology in Anesthesiology (STA)	1		5	
**International**					
	Abu Dhabi Health Services (SEHA)	1		54	
	Gateway Consulting, Singapore	27		395	
	Israel Ministry of Health	1		11	
	King Saud University (KSU), Saudi Arabia	4		83	

## Lessons Learned

My experience in developing all of the above educational offerings have resulted in many lessons learned. Although I ventured into online learning with little formal training, I discovered over time what works well for the kinds of teaching I prefer. Although academic lectures receive their share of criticism, I find that an engaging speaker can provide a highly effective learning experience, especially if he or she explains the big picture and fills in the necessary details. I know that PowerPoint also has its critics, yet it too can be highly effective, and perhaps more so in an asynchronous setting where lectures can be paused or reviewed. As such, my main teaching modality has always been lectures using voice-over PowerPoint slides. A typical 3 hours’ worth of lecture is segmented into 6-9 lectures. I also provide students with PDF handouts of the slides and an exhaustive list of references cited in the slides. The homework in the course consists of 10 multiple choice questions per unit. In the questions, I aim to require students to apply the material. The course does not have a required textbook, although I do tell students that it follows the contents of a textbook that I edited [16].

I have also learned that online teaching does not deserve what many perceive as lacking interaction. I have always taught from an LMS that featured discussion forums and advise students to think of such discussion forums as the online equivalent of a classroom. Students are encouraged to speak up, not feel intimidated, and remember that everyone has something valuable to say. In the introductory course, I seed the discussion with 1-2 questions but encourage students to also post their own questions, including asking about things they do not understand (rather than emailing me, to which my reply is usually to post their questions on the forum).

Another lesson learned is to advise students at the outset to follow some simple etiquette for the discussion forums. Messages should be neither too short nor too long. Everyone should be constructive and respectful. Students should reply to messages in their respective threads so that everyone can see the evolving discussion. Students should not copy and paste from websites but rather use their own words and provide a link if desired. They should also not discuss homework multiple choice questions until 1 week after the due date.

In addition, I have learned to lay out what I consider to be my expectations of students. They should complete the lectures and participate in the discussion. They should not be afraid to speak up in the discussion forums. They should ask questions about anything that is unclear in lectures or other materials. Most of all, they should feel free to challenge the instructor. Students should complete all assignments by the due dates. I do allow them to occasionally complete assignments late but warn them not to fall too far behind, since they will have difficulty in catching up.

By the same token, I tell students that they should have expectations for me. They should expect me to create an environment of learning and objective inquiry. I should maintain high availability, replying to emails as quickly as I can. They should expect that I am there to serve them, as students are not wasting my time. The best method of initial contact is email, and we can talk via video or phone as needed.

For lectures, students should expect the quality to be very good although not perfect. I am not a talking head and try to convey my view of informatics, getting into the details but never losing the big picture. One of my best compliments ever came in a course evaluation from a student who said, “I like that Dr Hersh pauses and makes mistakes and corrects himself … It shows he is thinking about what he is saying instead of reading off a paper” (even though I have more recently adopted using a script to make sure I cover everything on each slide). Students should also expect that in the discussion forums, I should read all postings even if I cannot reply to each post. I usually try to reply to threads where dialogue has developed and reply to different students and not the same ones each time.

An additional lesson learned is that online curricula, like any course curricula, need time and effort to be maintained. Fortunately, the large audience for my introductory course gives me the time and resources to keep the curriculum up-to-date. My basic approach is to update the course content once a year, refreshing the existing content and adding new material. This demonstrates a challenge for materials developed for posting online, such as the HITECH curriculum described above [12], that is, static content becomes outdated quickly. As such, the content soon diminishes in value for teaching.

Another lesson learned has been that my online materials prove very useful for in-person teaching and classes. This is typically done using a flipped classroom approach, where students listen to recorded lectures before coming to in-person class to discuss concepts, ask questions, give presentations, and more. Since moving my teaching to online lectures, it has been extremely rare for me to stand in front of a classroom and give a traditional lecture.

A final lesson learned is that online education is quite scalable. It requires a substantial fixed effort to develop and maintain content, although it has a very low marginal effort to provide that content to additional learners. Fortunately, the collection, curation, and maintenance of course materials is something I enjoy, even if the time commitment is substantial. However, once the content is produced, the additional work of developing new courses by repurposing it is modest.

## Conclusions

**Figure 3:**
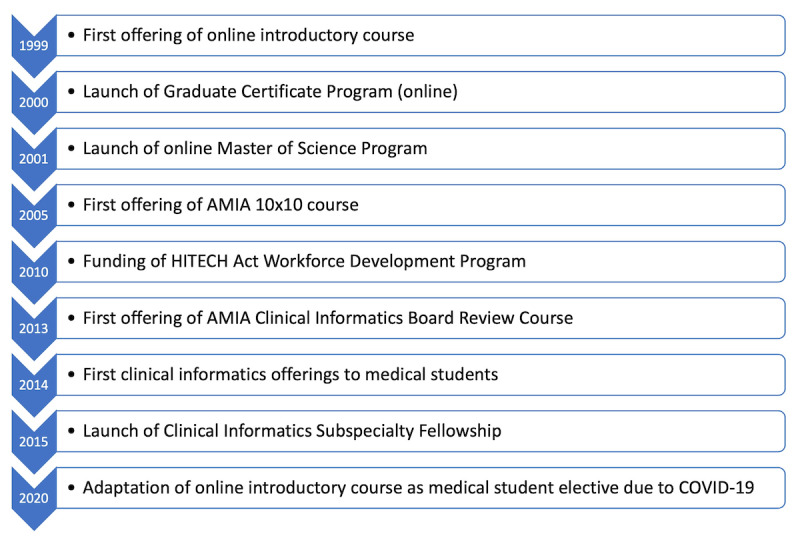
Major milestones by year. AMIA: American Medical Informatics Association; HITECH: Health Information Technology for Economic and Clinical Health.

The seemingly small decision to start offering my introductory informatics course in an online format had far-reaching implications for my academic informatics career. In the early days, many educational traditionalists scoffed at the notion of teaching online. But in modern times, education has had to adapt to different learners. Especially in knowledge-based fields, we no longer stop our education with obtaining a degree. Many professionals change direction as they move through their careers, with a common example being health care and other professionals moving into fields such as informatics. For many, the ability to learn online and asynchronously offers educational opportunities that cannot be met by traditional in-person classrooms. Figure 3 highlights the major course or program milestones by year.

## Abbreviations

AMIA: American Medical Informatics Association
ARM: Advanced Reduced Instruction Set Computing Machines
BCB: Bioinformatics/Computational Biomedicine
BMI: Biomedical Informatics
CIBRC: Clinical Informatics Board Review Course
DBMI: Department of Biomedical Informatics
HCIN: Health & Clinical Informatics
HIMSS: Healthcare Information and Management Systems Society
HITECH: Health Information Technology for Economic and Clinical Health
LMS: learning management system
MD: Doctor of Medicine
MINF: Medical Informatics
MS: Master of Science
NLM: National Library of Medicine
OHSU: Oregon Health & Science University

## References

[1] Hersh WR, Junium K, Mailhot M, Tidmarsh P (2001). Implementation and evaluation of a medical informatics distance education program. J Am Med Inform Assoc.

[2] Ash J, Hersh W, Krages K, Morgan J, Schumacher R (1999). The Oregon IAIMS: then and now. Bull Med Libr Assoc.

[3] Hersh W (2018). Methods Inf Med.

[4] Straus S, Glasziou P, Richardson W, Haynes R (2018). Evidence-Based Medicine - How to Practice and Teach EBM.

[5] Safran C (2018). Informatics training for clinicians is more important than hardware and software. Yearb Med Inform.

[6] Hersh W, Williamson J (2007). Educating 10,000 informaticians by 2010: the AMIA 10x10 program. Int J Med Inform.

[7] Otero P, Hersh W, Luna D, González Bernaldo de Quirós F (2018). A medical informatics distance-learning course for Latin America. Methods Inf Med.

[8] Feldman SS, Hersh W (2008). Evaluating the AMIA-OHSU 10x10 program to train healthcare professionals in medical informatics. AMIA Annu Symp Proc.

[9] Hersh W (2006). Who are the informaticians? What we know and should know. Journal of the American Medical Informatics Association.

[10] Hersh W, Wright A (2008). What workforce is needed to implement the health information technology agenda? Analysis from the HIMSS analytics database. AMIA Annu Symp Proc.

[11] Blumenthal D (2011). Implementation of the Federal Health Information Technology initiative. N Engl J Med.

[12] Mohan V, Abbott P, Acteson S, Berner ES, Devlin C, Hammond WE, Kukafka R, Hersh W (2014). Design and evaluation of the ONC health information technology curriculum. J Am Med Inform Assoc.

[13] Detmer DE, Shortliffe EH (2014). Clinical informatics: prospects for a new medical subspecialty. JAMA.

[14] Longhurst C, Pageler N, Palma J, Finnell J, Levy B, Yackel T, Mohan Vishnu, Hersh William R (2016). Early experiences of accredited clinical informatics fellowships. J Am Med Inform Assoc.

[15] Hersh WR, Gorman P, Biagioli F, Mohan V, Gold J, Mejicano G (2014). Beyond information retrieval and electronic health record use: competencies in clinical informatics for medical education. AMEP.

[16] Hersh W (2022). Health Informatics - Practical Guide, 8th Edition.

